# Indigenous Food Systems and Climate Change: Impacts of Climatic Shifts on the Production and Processing of Native and Traditional Crops in the Bolivian Andes

**DOI:** 10.3389/fpubh.2016.00020

**Published:** 2016-03-03

**Authors:** Alder Keleman Saxena, Ximena Cadima Fuentes, Rhimer Gonzales Herbas, Debbie L. Humphries

**Affiliations:** ^1^Department of Anthropology and School of Forestry and Environmental Studies, Yale University, New Haven, CT, USA; ^2^Fundación PROINPA, Unidad de Recursos Genéticos, Cochabamba, Bolivia; ^3^School of Public Health, Yale University, New Haven, CT, USA

**Keywords:** agrobiodiversity, Bolivia, Andean agriculture, indigenous food systems, climate change impacts, food security

## Abstract

Inhabitants of the high-mountain Andes have already begun to experience changes in the timing, severity, and patterning of annual weather cycles. These changes have important implications for agriculture, for human health, and for the conservation of biodiversity in the region. This paper examines the implications of climate-driven changes for native and traditional crops in the municipality of Colomi, Cochabamba, Bolivia. Data were collected between 2012 and 2014 *via* mixed methods, qualitative fieldwork, including participatory workshops with female farmers and food preparers, semi-structured interviews with local agronomists, and participant observation. Drawing from this data, the paper describes (a) the observed impacts of changing weather patterns on agricultural production in the municipality of Colomi, Bolivia and (b) the role of local environmental resources and conditions, including clean running water, temperature, and humidity, in the household processing techniques used to conserve and sometimes detoxify native crop and animal species, including potato (*Solanum* sp.), oca (*Oxalis tuberosa*), tarwi (*Lupinus mutabilis*), papalisa (*Ullucus tuberosus*), and *charke* (llama or sheep jerky). Analysis suggests that the effects of climatic changes on agriculture go beyond reductions in yield, also influencing how farmers make choices about the timing of planting, soil management, and the use and spatial distribution of particular crop varieties. Furthermore, household processing techniques to preserve and detoxify native foods rely on key environmental and climatic resources, which may be vulnerable to climatic shifts. Although these findings are drawn from a single case study, we suggest that Colomi agriculture characterizes larger patterns in what might be termed, “indigenous food systems.” Such systems are underrepresented in aggregate models of the impacts of climate change on world agriculture and may be under different, more direct, and more immediate threat from climate change. As such, the health of the food production and processing environments in such systems merits immediate attention in research and practice.

## Introduction

There is growing consensus that the world’s climate is changing (1–4). Though the timing, magnitude, and direction of such changes are likely to vary from one place to another, researchers predict that climatic changes will have important impacts on agricultural production (4–8), on the health and function of ecosystems, and on human health (9–11). Furthermore, these challenges are increasingly understood to be interrelated: human health outcomes are closely linked to the health of ecosystems and agricultural lands (12–14).

The projected climatic changes in the Andes have important ramifications for agriculture, biodiversity, and nutritional health. Observers have documented the retreat of glaciers throughout the region, a trend toward higher levels of precipitation in the inner tropical Andes, and drying in the subtropical Andes (15, 16). Under these circumstances, they predict a “massive warming” on the order of 4.5–5°C for the tropical Andes by the end of the twenty-first century (16). This scale of warming would threaten communities and watersheds relying on glacier-fed water supplies (17) and would have important consequences for agricultural production. Underscoring these projections, in Andean Bolivia, local reports based on farmers’ observations detail shifts in the weather and climatic conditions that are already being observed (18–20). A study in four of Bolivia’s departments, representing the major agricultural ecotypes, found that farmers in all regions perceived agriculture to have become riskier due to changing weather and climatic patterns (18).

Climatic changes in the Andes also stand to influence the conservation and use of this region’s reserve of globally important agricultural biodiversity. The Andes are considered a center of origin and diversity for potatoes, quinoa, amaranth, and many other minor crops (21, 22). Although Andean diets have changed to incorporate processed foods and improved crops (such as rice, pasta, and “improved” potato varieties), native crops still play an important role in household consumption in both rural and urban areas (23). Observers of agrobiodiversity in the Andes, as in other regions of the world, have hypothesized that a major function of the cultivation of diverse crop species and varieties is to make agriculture more resilient to risk (24, 25). The creative management of diverse species, varieties, and landscapes has been proposed as a tool-kit for adaptation to climate change in small-scale agriculture (26), though observers of the high-mountain Andes have also warned that severe climate impacts may push traditional systems beyond their adaptive limits (27).

Compounding these threats, Andean populations are often nutritionally vulnerable, with Bolivia exhibiting the highest levels of undernourishment in South America (28). Bolivia’s 2008 DHS survey reported that, at a national level, 27.1% of children could be classified as having low height for age (stunting) (29), though official national statistics suggest this figure has decreased to 15.9% in recent years (28). These problems are more acute in rural areas: in 2010, the World Food Program estimated that some 37% of rural Bolivian children under age 5 suffered from stunting and 59% of rural households earned an income insufficient to meet basic food needs (30). Given populations that are already vulnerable to food insecurity and malnutrition, climatic shifts affecting agricultural productivity and food processing technologies could have significant impacts on rural populations.

In this paper, we detail results of a case study of the impacts of climate change on the role of native and traditional crops (NTCs) in a local system of food production and consumption in the municipality of Colomi, Bolivia. To provide a foundation for the research reported herein, we provide conceptual background on “indigenous food systems,” Andean agriculture, and the role of locally developed processing practices in detoxifying and preserving NTCs. Subsequently, we present our research objectives and methods, and detail our results. Our data suggest that, with increasing uncertainty around weather and climate, household farming practices are changing, with new priority placed on a narrower range of crops and planting seasons that help to reduce combined environmental and market risk. We also detail the reliance of household processing practices on the availability and predictability of local environmental resources, which may be vulnerable to climatic shifts. We conclude with a discussion of the ramifications of these findings for the role of native crops in Bolivian food systems, and their implications for the study of indigenous food systems under climate change. This case reflects a larger issue of “dueling epistomologies” in academic and practitioner understandings of the relationships linking climate change, agriculture, and food security in indigenous vs. industrial food systems.

## Background: Andean Indigenous Food Systems and Climate Change

We use the term “indigenous food systems” to refer to systems of cultivation, processing, storage, trade, and consumption, which are specific to particular geographic regions, and whose origins generally pre-date large-scale industrial agriculture. In this sense, “indigenous food systems” would include systems relying primarily on minor and/or endemic food crops (including native or underutilized species), or farmer-saved varieties of major food staples, such as corn, rice, and wheat. Cultivation and processing techniques in such systems are primarily manual or non-industrial, though such practices are subject to change over time.^1^ Many examples exist of indigenous or small-scale agricultural systems which incorporate new technologies and techniques, or which engage with larger commercial and industrial markets (43–46). Nonetheless, when viewed in broad perspective, these systems differ, qualitatively and quantitatively, from mechanized industrial agricultural systems relying primarily on hybrid or formally “improved” germplasm of the world’s major food staples. In our use, the term also encompasses local practices of trade, marketing, and consumption, which may be undertaken in ways which reflect historical or contemporary relationships within and among ethnic groups, or other culturally specific patterns of symbolism, identity, and meaning-making. Notably, in our use of the term, the “indigeneity” of these systems is defined not by the ethnicity of the people who participate in them, but rather by the historical and cultural origins of the crops that they include, and the techniques that are used to plant, grow, harvest, process, and store such crops.^2^

High-mountain Andean agricultural systems of the type we discuss in this paper are an example of an “indigenous food system.” These systems rely on a diversity of crop species and varieties, and are a center of origin and diversity for multiple major and minor tubers, grains, and legumes, as well as tropical and subtropical fruit species (21, 22). They also house important mammal biodiversity in the South American camelids, including llama (*Lama glama*), vicuña (Vicugna vicugna), and alpaca (*Vicugna pacos*) (48, 49). In addition to species diversity, farmers have historically utilized landscape diversity as a tool, with households distributing their agricultural production practices across parcels of land located in different altitudinal ranges (50, 51). The complementarity of agricultural practices and production in different altitudinal zones holds true beyond the household level; even prior to European contact, Andean residents participated in patterns of large-scale, long-distance trade, in which groups of people migrated seasonally to trade their agricultural produce with members of kinship networks located in other agroecological regions. In Andean studies, this phenomenon is termed “verticality,” and the socio-spatial arrangement resulting from it is designated the “vertical archipelago” (52, 53).

Though Andean agricultural and food systems have changed to incorporate introduced crops and foods, these systems have proven resilient to changes wrought by industrial agriculture (43, 45). NTCs are prevalent in the diets of Andean residents, and foods such as potatoes, highland maize varieties, and minor tubers, play important roles in Bolivian festival and everyday dishes. Furthermore, the Andes are a center of contemporary human cultural diversity: in 2012, some 31% of Bolivian citizens identified themselves as a member of one of the 36 indigenous groups formally recognized by the country’s constitution (54). In 2001, approximately 65.8% of the adult population identified as ethno-linguistically indigenous (55). Contemporary cultural practices incorporate elements of both historical indigenous traditions and novel influences.

In Andean Bolivia, agriculture is already being impacted by climate change, as detailed in local-level reports on changing weather and climatic conditions observed by farmers (18–20). A study in four of Bolivia’s departments, representing the major agricultural ecotypes, reported that farmers had observed not only changes in the maximum and minimum seasonal temperatures, with a general tendency toward increase, but also greater dangers of unseasonable, crop-damaging frost (18). Similarly, production was threatened by increases in rainfall in tropical regions, and decreases in montane or arid regions, a phenomenon that farmers understood to be compounded by the effects of deforestation. In general, farmers in all regions perceived agriculture to have become riskier due to climatic factors (18). This study also pointed out that some impacts of climate change on small-scale agriculture in Bolivia may be poorly understood, since statistics are often collected around extreme, disaster-scale events, but less attention is paid to the cumulative effect of unpredictable precipitation or weather during “normal” years (18).

One aspect of indigenous food systems that has been understudied in the context of climate change is household food processing, particularly as related to the presence of noxious or toxic plant chemicals. Pre-industrial food systems based on NTCs provide many examples of humans’ efforts to avoid toxicity in plant-based diets. For example, farmers in Amazonia and African countries have developed complex processes for removing cyanogenic glucosides (a precursor to the poison cyanide) from manioc (*Manihot esculenta*) (56, 57). The Andean crop quinoa (*Chenopodium quinoa*) is also a case in point, containing bitter saponins, which must be removed by rinsing or scarification prior to consumption (58, 59). The detoxification of plant-based materials has also been posited to explain the phenomenon of pica, or the consumption of non-food materials, such as earth, clay, and ice (60, 61).

Such local processing is particularly important in Andean indigenous food systems. The crops and foods grown in mountain environments like the Andes evolved with intense high-altitude UVA-UVB radiation (62), and often with grazing or browsing mammals, represented in the Andes by native camelids, such as llamas, alpaca, and vicuña (62, 63). Such conditions may stimulate plants to produce secondary metabolites for self-defense, either against solar radiation or herbivory (62, 64). To thrive under these conditions, Andean farmers have used wind, water, sunlight, and cold to detoxify unpalatable or potentially poisonous crops and to preserve crop harvests over long periods, as discussed below. Recent studies have drawn attention to the capacity of native foods to be adopted by and persist in commercial or industrial agricultural food provision, pointing out that traditional processing practices may, in some cases, be expanded upon or substituted by technological innovations (65–68). However, the potential impacts of climatic changes on the household processing techniques that detoxify native crops, increase their palatability, or ensure their long-term storage, are as yet understudied.

## Research Objectives

This article draws on mixed-methods fieldwork in environmental anthropology carried out between 2012 and 2014. This research was designed to address the question: what role do NTCs play in household food security and contemporary food culture in urban Cochabamba, and the rural municipality of Colomi? The project was designed to collect data on the broader relationships linking food security to agrobiodiversity, including issues surrounding agricultural production, markets, preparation practices, and cultural preferences. During conversations with research participants, climate and weather changes emerged as important modifiers of the production, processing, and use of NTCs. Here, we present a synthesis of key results on the production, processing, and use of NTCs in the municipality of Colomi, Bolivia, as related to ongoing shifts in weather and climate.

### Research Methods

Qualitative, ethnographic fieldwork was used to characterize the production techniques and household culinary uses associated with NTCs. Fieldwork was carried out in collaboration with Fundación PROINPA (www.proinpa.org) a Bolivian agricultural development research foundation, and with support from the Biomedical Research Institute (Instituto de Investigaciones Biomédicas) of the Universidad Mayor de San Simón. Trained research assistants were fluent in both Spanish and Quechua. Observations collected by the first author were triangulated with the knowledge of the second and third authors, who each have 20 years of research and applied project experience in the region. The study protocol was reviewed and approved by the Yale University Institutional Review Board (protocol #1107008769) and community data collection methods were reviewed and approved by the Bioethics Committee of the Universidad Mayor de San Simon Department of Medicine in Cochabamba, Bolivia.

### Study Locale

**Figure 1:**
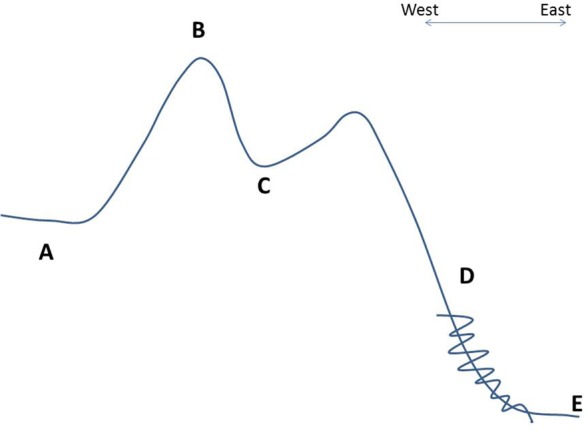
**Schematic of altitudinal gradient between Cochabamba and Amazonian Floodplain, with approximate altitudes (not to scale)**. **(A)** Urban Cochabamba, approximately 2700 masl; **(B)** puna (rural), approximately 4200 masl; **(C)** sub-puna (rural/urbanizing), approximately 3200 masl; **(D)** montane tropical zone (rural), approximately 2200 masl; **(E)** Villa Tunari (Amazon floodplain), approximately 400 masl.

The municipality of Colomi, located in the Department of Cochabamba, Bolivia, stretches over an altitudinal range of approximately 2200 masl to around 4200 masl (see Figure 1). This article focuses on crops grown in environments of 3000 masl and above, which can roughly be divided into a *sub-puna* region of up to 3600 masl, and the high-altitude *puna*, at 3600 masl and above. In Colomi, the sub-puna region is distributed around a valley bottom, which includes the fertile plain surrounding the Lago de Corani, a human-created reservoir. The high-altitude puna (hereafter “puna”), in contrast, largely consists of sloping lands or marshy, mountaintop plains.

### Data Collection

Results presented here are drawn from three different data collection streams: participatory workshops, participant observation, and semi-structured interviews (Table 1). From this data, we present two primary themes: the impact of climate change on agriculture in Colomi, and the role of environmental resources affected by climate change in the practices used to process and preserve native Andean foods. Data about the impact of climate change on agriculture in Colomi were collected primarily *via* participatory workshops, and authors’ cultivation of two native potato plots in 2013–2014, in collaboration with local community organizations. These data were supplemented by semi-structured interviews with agronomists familiar with the region. Information on practices used to preserve and prepare native Andean crops and meat for household consumption were collected primarily from participatory workshops, and supplemented by data collected *via* participant observation and semi-structured interviews.

**Table 1:** **Relationship between categories of data presented and field research methods**.

Data type	Participatory workshops with rural farmers	Participant observation		Semi-structured interviews with local agronomists
		Household food preparation	Potato cultivation	
Agriculture and climate change in Colomi	X		X	X
Native crop preparation practices	X	X		

#### Participatory Workshops

In 2014, a total of 10 participatory workshops were carried out across six communities: five communities in the rural municipality of Colomi and one community in peri-urban Cochabamba. The first workshop focused on seasonal dynamics of agriculture and food security (six communities), and the second workshop focused on household processing and consumption of native crops (four communities). The 6 sites were chosen as a sub-set of 10 sites where a larger survey project was being undertaken and were purposively selected to encompass altitudinal, ecological, and rural–urban variation. Women from these towns were issued an open invitation through radio announcements and local contacts to attend a workshop on agrobiodiversity, nutrition, and child health. Most participants were women with children under the age of 5, although some men and women with older children also joined. The workshops were bilingual (Spanish–Quechua) and included educational content and interactive games and group drawing activities, to better elicit local perspectives. Workshop participants were informed ahead of time that the workshops entailed both a research component and an educational component, and that data collected in the research component would be held in confidence. Data were recorded using photos, audio recordings, and detailed notes by a research team member. Notes, and supplementary materials such as drawings produced during workshop activities were digitally transcribed or photographed, as appropriate.

#### Participant Observation

Participant observation in household food preparation consisted of preparing and eating “typical” Bolivian meals, or dishes using NTCs, together with Bolivian key informants, colleagues, and research assistants. Participant observation by the first author took place opportunistically and repeatedly over the course of 19 months of in-country fieldwork. Participant observation in potato cultivation was undertaken as part of a larger project that sought to grow out a diverse set of potato varieties in two field plots under farmers’ conditions for comparison of their agricultural, morphological, and culinary characteristics. The first, second, and third authors participated in planting, care, and harvest of these fields, including discussions of management for the climatic conditions prevalent in the 2013–2014 agricultural cycle. Data on both forms of participant observation were recorded *via* detailed ethnographic fieldnotes and photos.

#### Semi-Structured Interviews

Conversations loosely structured around the themes of agriculture, food security, and food processing were undertaken with more than 20 Bolivian agronomists with experience in Cochabamba and Colomi. Although some agronomists were interviewed only once, the first author collaborated closely with a core group of seven individuals, with experience in the municipality ranging from 2 to 25 years, who were interviewed on multiple occasions over the course of fieldwork. Some interviews were audio-recorded, but many were documented *via* hand-written notes, both of which were subsequently digitally transcribed.

#### Data Analysis and Management

Qualitative field research methods were chosen for this project because they allow research participants to share experiences, practices, and knowledge on their own terms. As described by Bernard, qualitative data, “make *process* clear, and bring out subtleties in behavioral complexes” (emphasis added) (69). Additionally, qualitative methods have the advantage of allowing novel observations to emerge iteratively and inductively over the course of research, such that the researcher may come away with knowledge about a pattern or process to which he or she had not previously assigned importance (70, 71). Data were transcribed and thematically coded using the ATLAS-TI software (v. 7.5). The topics presented here emerged from themes identified for analysis by the primary author, and subsequently triangulated with coauthors.

## Results: Climate Change, Agricultural Production, and Native Crop Processing

### Weather and Potato Agriculture in Colomi, Bolivia

Farmers in Colomi plant at least five species of potato, the major staple crop, including *Solanum tuberosum* (sub-species *tuberosum* and *andigena*), *S. stenotomum*, *S. goniocalyx*, *S. juzepczukii*, and *S. phureja*. Colomi farmers have historically planted multiple varieties of each of these species, and for this reason Colomi has been considered a “microcenter” of potato diversity within Bolivia (72).

Colomi’s geography makes for a particularly unique and variable agricultural environment. The municipality spans the eastern lip of the Andean range, and a large part of the sharp downhill slope that becomes the tropical lowlands of the Amazon basin (Figure 1). Due to its orientation toward the windward slope of the mountains, the region receives a great deal of rainfall, resulting from the condensation of moist air rising up the tropical montane Andean slopes. This precipitation varies significantly by season, with the bulk of rain falling in the period between November and April. Dry weather conditions occur between May and October/November.

Within Colomi, mountainous topography creates the conditions for distinct microclimates, which may vary at a few kilometers’ distance. Towns on the eastern edge of the high-altitude puna are particularly affected by their exposure to the full force of windward slope rainstorms, thunderstorms, and hail. Meanwhile, high-altitude towns farther removed in mountain valleys may exhibit more consistently moist conditions, experiencing a tradeoff between fog and sunshine, and some protection from violent weather. Communities in the *sub-puna* are, in some cases, also protected by the topography of surrounding hills. Although temperatures rarely fall below freezing, except at higher altitudes, hailstorms are common in the rainy season, as are cold rain and winds.

Colomi farmers pay close attention to the weather, which can change rapidly. Rain squalls sometimes begin with little warning, meaning that farmers must perform moisture-sensitive tasks with urgency. For example, if rainfall catches farmers by surprise during the potato harvest, this can threaten the storage of potatoes both for seed and for the coming year’s consumption, because potatoes stored when wet are susceptible to rot.

### Potato Agriculture and Timing of Rainfall: Historic vs. Present Patterns

Historically, key informant agronomists report that there were as many as four distinct planting seasons for potatoes in Colomi (Table 2). In the *loqrum* season, around the month of February farmers planted precocious (short-duration) varieties typically requiring <3 months from planting to harvesting. Farmers also planted precocious varieties in the *mishka* season, in July–August. In the *chawpi mishka*, farmers planted longer*-*duration varieties with the first onset of the year’s major rains. Finally, in the *jatun tarpuy* (meaning “big planting” in Quechua), farmers planted the bulk of their potato crop for the year, with the full onset of seasonal rainfall in October and November.

**Table 2:** **Historic planting periods in Colomi, with approximate harvest seasons**.

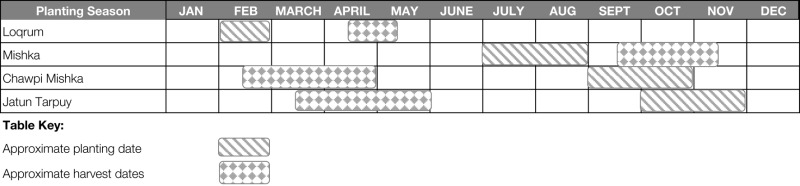

Agronomists who served as key informants noted a marked change in the rainy season over recent decades. Historically, there was enough rain in the months of July–August to reliably begin planting in these months (i.e., during the *mishka* or *chawpi mishka*). However, in years prior to the fieldwork reported here, the peak rains in Colomi were delayed, typically observed in late September, October, or even November. Additionally, in more recent years, agronomist participants report a shift in the distribution of rainfall. Agronomists observe that rainstorms now often deliver short, heavy bursts of precipitation, rather than more gentle episodes, as might have been the case 20–30 years ago.

Current weather patterns reported by farmers, outlined in Table 3, correspond with agronomists’ observations. In three of the five rural sites where participatory workshops were carried out, farmers reported rainfall starting only in the month of November. The two sites where rainfall was reported earlier, Corani Pampa and Pico Central, are located in tropical and protected valley microclimates, respectively, which offer some buffering from the region’s larger weather patterns. Among these five sites, dry or drought conditions are now reported in the months of July to October, which would historically have been important periods for planting potatoes.

**Table 3:** **Current annual weather patterns reported in five rural study sites in Colomi, Bolivia**.

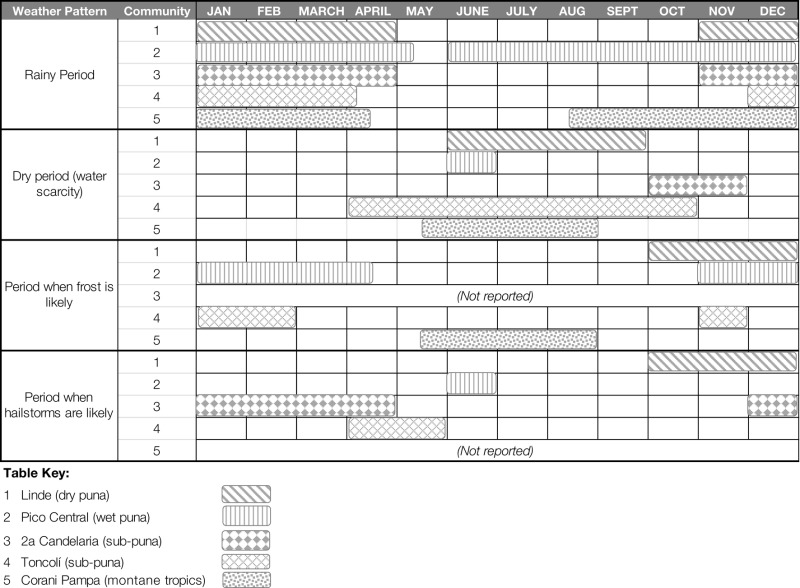

Farmers also report a likelihood of exposure to frost and hailstorms, which can damage the potato crop, at certain times of year (Table 3). Generally, at higher altitudes, frost was reported to be more likely during the rainy season period of November–April. In the lower-altitude montane tropics, farmers perceived the risk of frost to be greater in the period between May and September, possibly reflecting differing weather patterns. Farmers’ identification of the likelihood of hailstorm follows a pattern less closely identified with altitude, possibly reflecting the influence of specific microclimates.

In response to these changes in the timing and distribution of rainfall, most Colomi farmers have adopted the strategy of planting just once a year, with the heavy-onset rains of the *jatun tarpuy*. Farmers reported planting in a single block of time, stretching from as early as June through the month of November (Table 4). In four of five sites, farmers reported planting during a period of only 3 months duration. Notably, the site where farmers reported planting over a longer period, Segunda Candelaria, is located in a geographical region allowing farmers easy access to land in both the *sub-puna* and the higher-altitude slopes of the *puna*, possibly allowing farmers to access a wider range of growing environments in a single season.

**Table 4:** **Approximate timing of potato planting activities reported in five rural sites in Colomi, Bolivia**.

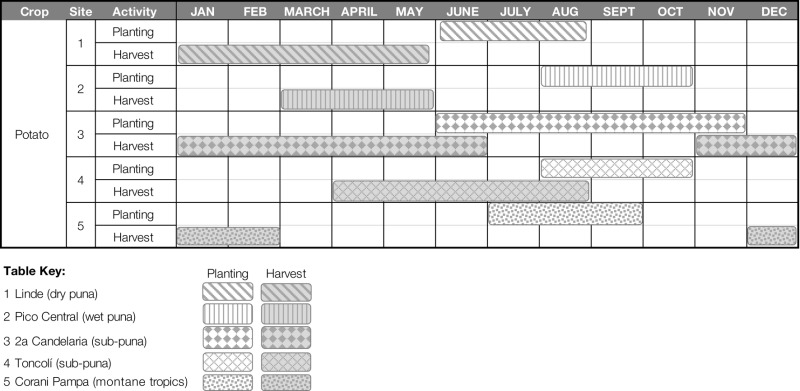

In considering the effects of changes in the pattern of rainfall on the timing of planting, it is important to acknowledge that Colomi farmers plant not by the calendar, but in direct response to their observation of rainfall, which guides soil preparation. Potatoes should not be planted when soil is entirely wet, so farmers must observe a good rainfall, then wait for a few days for the soil to drain, and finally plant seed quickly before another hard rain occurs. As a case in point, in the 2013 agricultural season, heavy rains began earlier than expected, in September and October, forcing many farmers to rush to prepare their land and seed for planting. Observers worried that the earlier onset of the rain would be coupled with another pattern observed in recent years, i.e., a relatively dry period in the following February–March which, if it coincided with crop flowering, could have disastrous implications for the year’s harvest.

### Soil Management, Potato Blight, and Native vs. Commercial Varieties

Locally, farmers and agronomists have historically divided soil into at least three types: *phurumas*, referring to virgin soils, or soils that have recently been plowed after a multiple-year period of fallowing; *ideáceas*, a term referring to soils that have been planted for the past 1–2 years; and *kutiyus*, a term referring to soils that have been used for a period of time, and are now in need of fallowing. One agronomist remembered a time, in the early 1990s, in which Colomi farmers paid detailed attention to the specific kind of soil in their plots and would leave a 5-year period of fallow between plantings.

Recently, however, agronomists observe that fallowing times have typically decreased to 1–2 years. One agronomist related to this decrease in fallowing time with an increased tendency to produce improved varieties of potatoes for commercial markets, a phenomenon he also associated with increased agricultural pest pressure. This individual observed that soils in the fertile valley bottom of the sub-puna had recently become more clay-like (*arcillosas*), perhaps due to a reduction in organic material after multiple periods of planting without fallowing. These circumstances left farmers more vulnerable to infestations of the fungus *tizón temprano* (*Alternaria solani*, or early blight). Rather than planting early in the season, which would help to reduce the vulnerability of young plants to pest pressure, farmers planting high clay-content soils with lower capacity for water retention found it necessary to wait until later in the rainy season to plant potatoes. Since the rainy season now frequently arrived later than usual, this meant that these farmers were more likely to be exposed to crop losses following late planting.

These changes are related to the types of varieties that farmers choose to plant. Improved varieties of potatoes are considered by farmers and agronomists to give higher yields in un-fallowed soils than native varieties. Meanwhile, native varieties are considered to no longer yield well in the soils of the sub-puna, instead requiring freshly plowed *phurumas* for good production. Furthermore, farmers locally consider improved potato varieties to be far more resistant to *tizón tardío* (late blight, Phytophthora infestans) and other fungi and pests than native varieties. This perception was supported by our own analysis of susceptibility and yields from the cultivation of two potato plots in 2013–2014 (data not shown). As a result of these pressures, the cultivation of native potato varieties is increasingly geographically circumscribed to higher-altitude areas of the *puna*, where fields are often far removed from transportation, and more steeply sloped. These characteristics, combined with lower population density, make such lands less suitable for intensive, commercially oriented agriculture, and more likely to be allowed longer fallowing periods, making the puna suitable for native potato varieties.

Potato varieties that are locally considered to be pest resistant, not incidentally, are largely commercial varieties. Commercial varieties include both formally improved varieties, such as Desiré (*papa holandesa*, or *Dutch potato*), and “native commercial” varieties, which have not undergone formal improvement by crop breeders (73). Rather than planting primarily for subsistence, many farmers prioritize the production of commercial potatoes (both native and improved) for sale, and subsequently supplement their household consumption with market-purchased goods such as pasta and rice. These practices are facilitated by the fact that the municipal seat of Colomi, located on the main highway linking the cities of Cochabamba and Santa Cruz, is a major wholesale market for agricultural products. Colomi farmers have relatively easy access to this twice-weekly market outlet.

### Native Crop (and Animal) Processing in Colomi

Results in the previous section outlined some ways in which potato agriculture in Colomi has already responded to changing environmental conditions. In this section, we present results of observations of native crop processing practices, which demonstrate the reliance of processing techniques on environmental resources, which in turn are linked to climate and weather. This reliance may indicate potential future impacts of climate change on the local food system.

**Figure 2:**
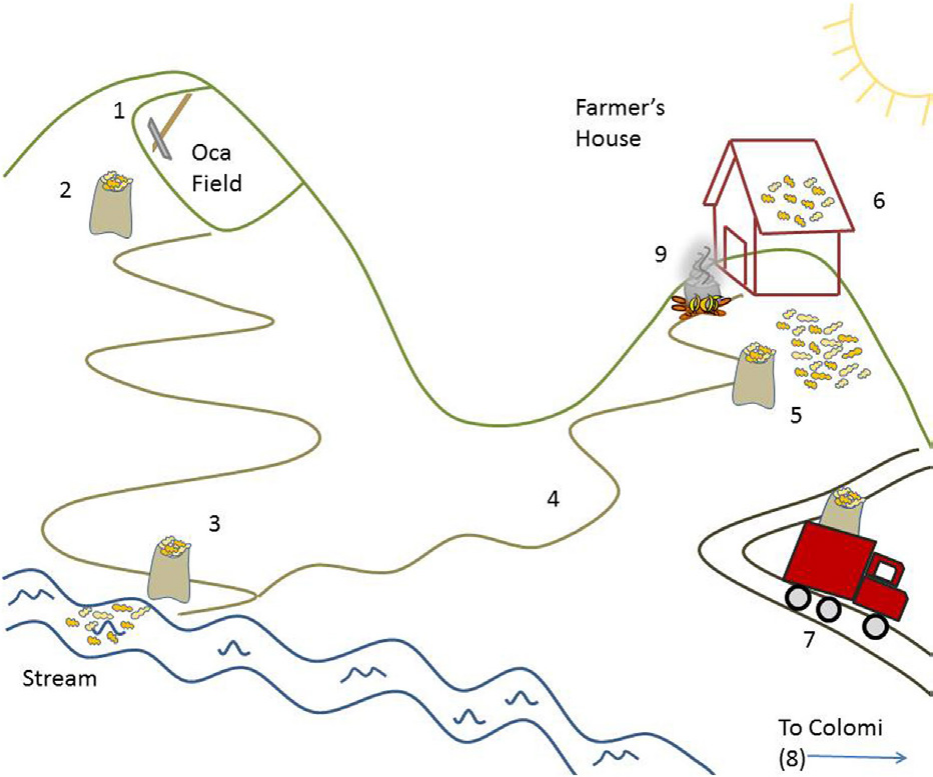
**Spatial schematic of Oca harvest and processing from participatory workshop in Colomi, Bolivia**. (1) Oca tubers are dug from field with pick-axe (*piquete*); (2) harvested tubers are bagged in plastic burlap sacks (*gangochos*); (3) Oca tubers are transported from field to a running water source (stream or river) for washing; (4) Oca tubers are transported to farmer’s home; (5) tubers are spread on ground to dry and sorted by size; (6) small tubers selected for immediate consumption are left spread to sweeten in the sunshine (on the ground or the rooftop) for up to a week; (7) Oca tubers selected for sale are transported to market on local roads; (8) Oca tubers are sold in twice-weekly market in Colomi municipal seat; (9) tubers not selected for market are prepared for home consumption.

**Figure 3:**
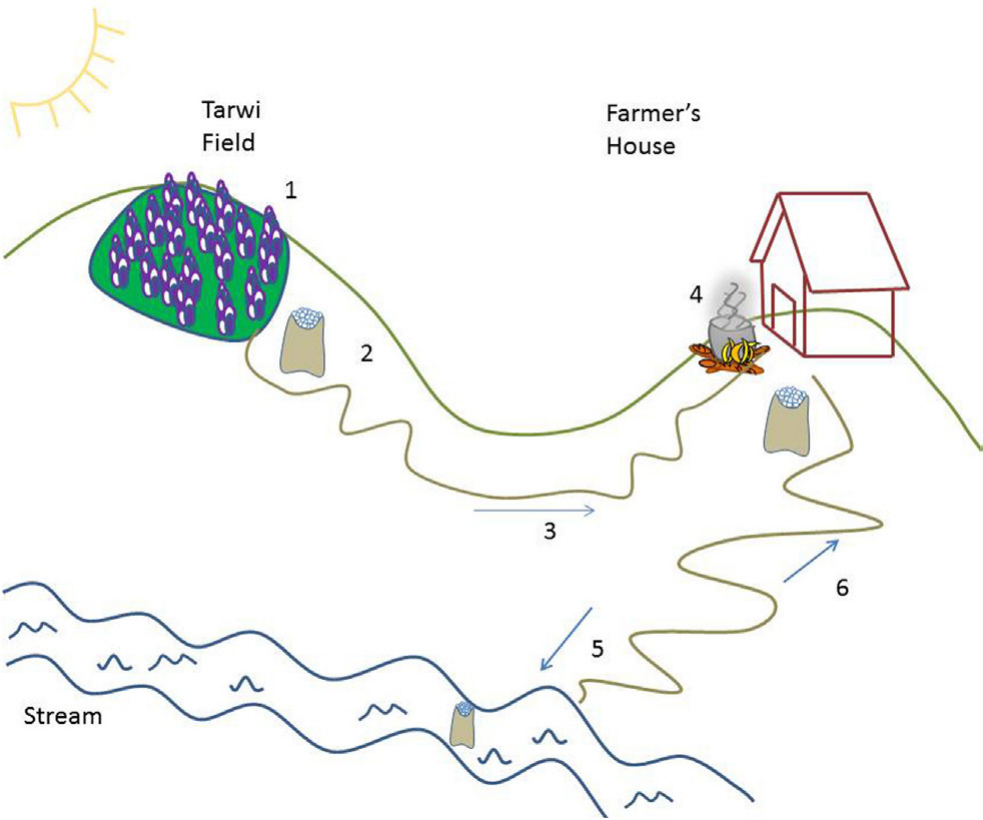
**Spatial schematic of tarwi harvest and processing adapted from material generated in participatory workshops in Pico Central and Linde/Pisly, Colomi, Bolivia**. (1) Tarwi plants are left in the field and are allowed to dry in the sun. (2) Tarwi grain is threshed, allowing the wind to remove the husk; thin or green grains are removed; and good grain is gathered in bags. (3) Tarwi bags are transported to household. (4) Tarwi is boiled in home kitchen. (5) Boiled tarwi is taken to the river in by *cuartillas* (smaller volumes) and left to rinse for up to 1 week. (6) Tarwi is consumed directly as boiled grain (*mote*) or made into a soup (*aguado*).

In participatory workshops discussing native crop processing with female farmers/heads of household, participants and facilitators produced schematic diagrams of the way that different aspects of the landscape between the field and the household were used to prepare crops for storage, consumption, and market (Figures 2 and 3). As the sections below highlight, these locally based processing techniques all rely to some extent on access to and reliability of environmental resources, such as water, daytime and nighttime temperatures, and humidity (Table 5). Specific details of these processing practices are outlined for four native crops (potato, oca, papalisa, and tarwi), along with two processing techniques that can be applied to multiple crops or species (*chuño* and *charke*).

**Table 5:** **Environmental resources used for processing and preparation of locally produced foods in Colomi, Bolivia**.

		Environmental resources			
Crop/breed	Product	Cold running water	Sunshine/daytime warmth	Low humidity	Nighttime freeze
*Potato*	*Tuber*	X			
	*Chuño*	X	X		X
*Oca*	*Tuber*	X	X	X	
	*Chuño*	X	X		X
*Tarwi*	*Mote*	X			
*Papalisa*	*Tuber*	X			
*Sheep/llama*	*Charke*		X	X	

### Potato

Potato (*Solanum* sp.) is the most important staple for farm households in Colomi. Potato tubers are harvested by digging. Unlike other tuber crops grown in Colomi (see below), potatoes are *not* washed immediately following the harvest, because the introduction of moisture can induce rot. Seed potatoes are often stored in the field under a covering of straw or dried grass. Potatoes intended for consumption or sale are loaded into large plastic mesh sacks (*gangochos*), which hold about 100 kg of weight. These are transported to the household of the farmer, where they may be selected according to size and variety for specific consumption purposes.

Potatoes, like other tubers, are particularly sensitive for consumption and storage due to their limited period of dormancy. Some varieties of potatoes can be stored up to 9–12 months in cool, dark, dry conditions, with little air movement, which allow the tuber to remain dormant. Others, like the short-duration *phureja* varieties, have little to no period of dormancy, and must be continually replanted to replenish both edible tubers and seed. The need for careful management of potato tubers is compounded by the fact that sprouting potatoes are toxic due to the presence of alkaloids, and particularly the glycoalkaloid solanine, in the tuber’s tissue. Green, sprouted potatoes have a bitter taste and, while in small doses they may only cause a stomachache, in larger doses they are poisonous (61). Thus, maintaining adequate moisture and light conditions during harvest and storage is crucial to their post-harvest longevity.

Although there are many ways of categorizing potatoes, Colomi farmers use a cross-cutting categorization of “*aguachenta/aguanosa*” (watery) and “*arinosa/arenosa*” (floury), to distinguish culinary use. Large, flat potatoes, like the improved variety, *Desiré*, are generally categorized as *aguachenta/aguanosa*, because of their high water content. Food preparers consider these types of potatoes most appropriate for preparing French fries or chips (*papa frita*). Colomi farmers produce these potatoes in large quantities, but they are largely destined for commercial markets.

For daily, home consumption, *arinosa*, or floury/grainy potatoes are preferred. This category includes both varieties that are peeled prior to consumption, and those that are prepared as *papa wayk’u*, a term referring to tubers boiled with the skin intact. Varieties typically peeled include “native commercial” varieties, like *Waych’a;* improved varieties like *Robusta*; and less commercial native varieties, like *Yuca Papa* or *Runa Papa*. These types of potatoes, which tend to have large tubers and a thick, dense consistency, are prepared first by washing the dirt off of the skin, then peeling, and finally boiling. Potatoes typically served as *papa wayk’u* are native potato varieties, which have knobbier textures less amenable to peeling. These include a varietal group called *Q’ollus*, and the recently commercial varieties *Pinta Boca* and *Candelero*. After washing, these varieties undergo little preparation beyond boiling, ideally with a pressure cooker. They are served with the skin intact, either on their own or alongside another component of a meal.

### Chuño

The Quechua term *chuño* refers to potatoes, and sometimes other tubers, which have been desiccated for long-term storage using an indigenously developed freeze-drying technique (74–77). To make *chuño*, in the cold season of the year, potato tubers (preferably small-sized) are spread across the ground and are left in the open air for 2 days and 2 nights. This process allows them to freeze and thaw and leads to the buildup of fluid in the tuber’s tissue. At this stage, the intermediate product, fermented tubers called *g’acha chuño*, is sometimes consumed. Although the liquid is somewhat caustic, causing a burning sensation when it touches the skin, *g’acha chuño* has a light flavor and a delicate texture and is often added to soup. To finish the *chuño*-making process, the liquid built up in the first period of freezing is removed from the tubers using the weight of one’s body, by stomping or standing on them. The pressed tubers are then taken to the river to be washed, and left for one night or more. Subsequently, they are collected and again spread across the ground to be dried for up to 1 week, protecting them from rainfall. Colomi farmers report storing the final, dried product for up to 2 years. After reconstituting by soaking, it can be used in a wide variety of dishes, including soups, *phuti* (a mixture with eggs or potato), and *samay* (a hearty midday meal).

In the high-altitude *puna* site of Linde, farmers specified that the ideal time of year for making *chuño* fell in the month of June and July, a period corresponding to reliably cold temperatures and low levels of precipitation (Table 6).

**Table 6:** **Ideal periods for the preparation of chuño and charke, as reported in Linde, Colomi, Bolivia**.

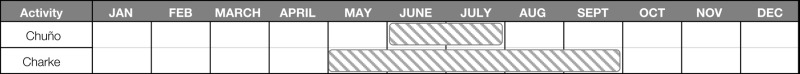

Dried *chuño* has an earthy flavor and a fibrous texture, quite different from the starchy flavor and smooth texture of fresh potatoes. Colomi farmers report making *chuño* to sell in markets as well as for home consumption. It is widely available in urban markets. Historically, *chuño* was made from small, bitter potato varieties, often of the species *S. juzepczukii* and *S. curtilobum*, referred to in Quechua as *luk’is*. Local farmers do not consider *luk’i* potatoes to be palatable when boiled as *papa wayk’u*, but they perform well at high altitudes, and are considered more palatable when prepared as *chuño*. However, with the decrease in cultivation of native potato varieties at lower altitudes, some farmers now make *chuño* from the smaller tubers of other native and native commercial varieties. *Chuño* may also be made from oca (see below).

### Oca

Oca is the tuber of the annual species *Oxalis tuberosa*. It is harvested by digging (see Figure 2), and collected in *gangochos*. Subsequently, families transport the tubers to nearby streams, immersing the tubers in running water to remove dirt. Once washed, they select the tubers by size. Large tubers are set aside for market, whereas smaller tubers are kept for home consumption. The smallest tubers are left for feeding livestock. Oca must be left out in the sun (*soleado*) prior to cooking, generally by spreading it across the ground, or the roof of a house, for up to 1 week. This is done to make it take on a sweet flavor, though researchers have also found that sunning decreases levels of oxalate, a chemical found in oca, which impedes calcium absorption and may be a precursor to kidney stones (78). This process is ubiquitous and is carried out for both rural and urban oca consumption. In urban markets, vendors sometimes sell oca which they advertise as “pre-sunned,” but it is also common to see baskets of oca sunning on front porches or yards of urban neighborhoods. Once oca tubers have been sunned, they have a reduced shelf-life and are subject to rot and mold.

Oca is usually served like a vegetable in soups or other preparations. Varieties of oca available in Colomi range in color from yellow to orange to red, and the flesh is usually yellow, with a flavor similar to sweet potato or carrot. Some participants reported preparing oca like they might prepare a native potato, either baking it or boiling it, and serving the cooked tubers whole as an accompaniment to a meal. Others consume oca as an ingredient of other preparations, such as soup, or in a dish called *oca samay*, which mixes fresh potato, fava beans, and potato *chuño*. Like potatoes, oca can also be freeze-dried as *chuño de oca*, using a similar process to that described above. Historically, this dried oca might have been ground into flour, which would then be used to make oca fritters (*buñuelo de oca*). However, agronomists and farmers who have observed oca production in Colomi over the long-term suggest that this form of preserving oca is becoming less common. Only one variety of oca exhibited the ideal characteristics for *chuño* production, and this variety is now far less frequently planted.

### Tarwi

Tarwi (*Lupinus mutabilis*) is an Andean grain related to the North American and European lupines. It is harvested for its protein-rich, leguminous seed, which grows at the end of long stalks. Farmers in workshops specified that, at the end of the season, it is allowed to dry in the field, after which it is harvested, removing the pods from the stem of the plant with a stick. It is then threshed to remove the pod, and put into bags, which are taken home for dry storage (see Figure 3). The grain is infrequently sold at market. In Pico Central, farmers report that it can be stored for 6 months. Tarwi is most frequently eaten as *mote*, a term which describes a seed that has been boiled long enough to be soft and is then eaten by hand. Research participants in Pico Central suggested boiling tarwi grain for an hour and a half, while research participants in Linde/Pisly reported putting three to four grains of wheat in the pot, and boiling the tarwi until the wheat grains were “popping” (*reventando*). After boiling, the tarwi grain should be taken to the river and left in a bag in the water to rinse for 1 week. Following rinsing, it can be eaten, and with the husk removed, it can also be prepared as an *aguado* (soup or drink). After undergoing this extensive preparation process, *mote de tarwi* (or *ch’uchusmuti*, in Quechua) maintains a bland and slightly bitter flavor, similar to boiled soybeans. When consuming, people generally remove the husk (which is still tough) and eat only the soft seed inside. *Mote de tarwi* is often sold along highways as a snack for passengers in cars or long-distance buses. Prior to processing, tarwi has a bitter taste due to high alkaloid content (79). According to research participants, when undercooked, tarwi has a reputation for being hard on the stomach, and provoking flatulence.

### Papalisa

Papalisa (*Ullucus tuberosus*) is a small, irregularly round, brightly colored tuber. Mottled patches on the skin range yellow to bright pink. In workshops, farmers reported that papalisa is generally planted in November and can be harvested as early as March, although the crop has a long growing cycle and may be left in the ground through November or December of the following year. At the end of the growing season, papalisa is harvested by digging, like other tubers, and is then taken to a river or stream for washing. In Colomi, papalisa production is largely oriented toward commercial markets, especially for the preparation of dishes consumed around the Easter holidays (*semana santa*). Farmers select round, healthy tubers for market sale, and keep tubers with a more oblong shape (*chhuqus*) for home consumption. To prepare papalisa for home consumption, it must first be boiled, ideally with salt. Subsequently, it is rinsed, to remove the skin and a slimy liquid (*baba*) produced by the tuber. The product may then be used to prepare in many dishes, including soup, as well as dishes such as *phuti de lisa*, *saqta de lisa*, and *lisa samay*. The latter are complex, flavorful, and heavier preparations (*secos)*, which may also incorporate vegetables, potato, and meat, egg, or peanut. They are cooked over both wood fire and gas. In home storage, fresh papalisa may be kept for 4–6 months. Cooked, the tuber is moist (not starchy) and has a distinctive flavor, similar to beets. Some research informants consider it to be an aphrodisiac.

### Sheep and Llama *Charke*

Particularly in the high-altitude *puna* town of Linde, participants reported making *charke*, a dried meat jerky, from cows, llamas, and sheep. For this purpose, a household might slaughter three to four animals a year. In focus groups, respondents suggested that this could take place at any given time of year, but while making annual calendars, research participants in the high-altitude site of Linde specified that the best time for making *charke* fell in the months of May to September, a period of the year with reliably dry temperatures, sun, and wind (Table 6). By contrast, in Pico Central, a site located in a humid, foggy valley, farmers reported not making *charke* at all because weather conditions do not allow for meat to fully dry. To make *charke*, farmers slaughter the animal, and on the same day or the day after, they cut the carcass into small pieces, including the bones. Meat is cut in strips of approximately the width of a human finger. Subsequently, each strip of meat is covered with salt, and hung from a wire, open to the elements, for 3 days. Once dried, it can be stored in a large, breathable plastic bag (*gangocho*). Respondents reported that *charke*, once prepared, can last more than 1 week, even if served in the morning, at lunch, and in the afternoon. In Linde, *charke* was not reported to be prepared for market sale, but rather was shared with nearby family members. When served in households, *charke* is often soaked in water, and sometimes fried in oil. Alternatively, it may be served in soup. Although respondents did not report selling *charke*, it is widely commercially available in urban Cochabamba. Much of what is available is beef *charke*, which comes from the Amazonian lowlands, but *charke de llama* is increasingly available in urban settings as well.

## Discussion: Indigenous Food Systems and Climate Change

### Impacts of Climate Change on Native Crop-Based Food Systems in Colomi, Bolivia

In Colomi, farmers and agronomists are already observing changes in the timing and distribution of rainfall, which they identify with the broader patterns of climate change. These changes have implications for the production and consumption of native and traditional Bolivian crops, and for these crops’ contribution to food security and nutritional health. Potential pathways of impact are summarized in Table 7.

**Table 7:** **Pathways of potential climate/weather change impacts on the production and consumption of native and traditional crops in Colomi, Bolivia**.

Mechanism	Cause	Potential outcomes
Reduction of yields	Increased exposure to drought or pest/disease pressure, especially at lower altitudes	• Increase in relative cost of native potato cultivation vs. commercial varieties • Increase in relative market price of native potatoes, vs. commercial varieties
Reduction in number of yearly harvests	Decreased predictability of onset and duration of annual precipitation	• Decreases number of harvests available per year • Decreases role of precocious (short-duration) native varieties in household consumption • Implies crops sourced in market from other regions (rather than locally produced) must be used to smooth consumption in periods of scarcity
Increased difficulty of local processing	Decreased availability or predictability of environmental resources (water, temperature, humidity) required for local processing techniques	• Potential for increased toxicity or spoilage, or reduced palatability in absence of conditions for traditional processing (potato, tarwi, oca, chuño) • Reduced post-harvest storage of perishable preparations (chuño, *charke*)

Among these pathways, farmers and agronomists already perceive patterns in the impacts of changing weather patterns on the viability of native potato varieties. Chief among these impacts is an observed shift in the timing of rainfall, driving a change in farmers’ planting and harvesting strategies. Farmers have reduced the number of planting seasons from two or more to one per year, effectively reducing the number of harvests available to a household, and increasing the length of time between them. This strategy also stands to increase households’ vulnerablity to the loss of any single harvest, removing the buffer of another recent or anticipated harvest in the short-term.

In addition to the observed reduction in the number of planting seasons, local agronomists hypothesize that a reduction of fallowing times changes the quality of the soil, and that this tendency combines with changes in weather patterns to increase crops’ exposure to agricultural pests and diseases. In potatoes, this process marginalizes the use of native or local varieties, because commercial varieties of potato tend to be more resistant to pests. Farmers restrict their planting of native varieties to recently fallowed, higher-altitude conditions, where pest loads are lighter, while reserving more fertile and accessible lowland plots for commercial varieties. This process may function in a feedback loop with population growth, coupled with households’ increasing integration with market and government institutions, which require cash for participation.

Although research participants already perceive impacts of changes in climate and weather patterns on potato production, an analysis of the role of other climate-affected environmental resources in traditional crop processing systems identifies potential vulnerabilities in this aspect of NTC consumption as well. As summarized in Table 6, local processing techniques, which remove bitter plant toxins and make native crops and livestock amenable to long-term storage, rely heavily on the availability of cold, running water; the predictability of daytime warmth or sunshine and nighttime freezing temperatures; and reliable levels of humidity. Under current climate predictions, these environmental conditions may be under threat. For example, if water shortages occur, farmers might simply not have access to the water necessary to process their crops. This would have a basic effect on households’ ability to consume all tuber crops, which must be washed prior to preparation. Furthermore, it might severely limit farmers’ ability to prepare *chuño* and *tarwi*. The latter requires multiple days of rinsing in running water to be made more palatable, and the former requires a similar process both to remove bitter flavors and to prepare it for long-term conservation.

Similarly, the predictability and reliability of temperature and humidity are major factors enabling or constraining farmers’ ability to process crops and livestock, and ensure post-harvest storage. If temperatures do not fall low enough at night, or rise high enough in the day, the processes employed to make *chuño* would otherwise simply result in rotten potatoes. This limitation has already been observed in the Bolivian *altiplano*, where some communities report that changes in the timing and predictability of freezing nighttime temperatures has made it difficult for them to produce *chuño* (80). Similarly, under humid or foggy conditions, meat prepared as *charke* may spoil, a phenomenon reported to be a deterrent to *charke* production in the research site of Pico Central. In the case of oca, without adequate sunlight, farmers might simply be exposed to higher loads of toxins and have to consume a less sweet (and therefore less palatable) product. Though potatoes undergo little processing, exposure to sunlight, warmth, or heat during the storage period may trigger them to exit the dormant phase, increasing the presence of toxic alkaloids in their tissues. Meanwhile, exposure to moisture may simply provoke decay (81).

Temperature and humidity are not commonly thought of as “environmental resources.” However, in this context, the key characteristic of these resources is their *predictability*, which in turn influences farmers’ sense of environmental risk. Because the household preparation and preservation processes described here require time, farmers do not have the option of waiting to see how low the temperature falls on a given night before spreading potatoes to make *chuño*, or waiting to see if it is a particularly dry (or humid) day before making the decision to slaughter a llama for *charke*. Rather, these decisions must be made based on their anticipation of future conditions, drawing from recent past experience. As such, the unreliability of environmental conditions may threaten local food processing systems not only through actual losses of food but also through a perceived increase in the riskiness of engaging household processing activities. This may, in turn, reduce farmers’ willingness to engage in the production and consumption of native crops.

### Industrial Processing for Native Crops: A Viable Alternative?

The observations outlined above apply particularly to the production and processing of native crops for household subsistence. However, the municipal seat of Colomi, located on the major highway that connects La Paz to Santa Cruz, is a market hub (73), and as such most farm households regularly access markets as both buyers and sellers. The availability of commercial markets in this region may be presumed to offer a buffering function for food security, providing the option to purchase food if a crop fails or to engage in wage labor to earn money. However, the extent to which such markets might offer substitutes for household-level production and processing of agrobiodiversity is uncertain.

Could local processing systems be substituted or replaced by industrial processing for market sale? As outlined above, the NTCs and meat products produced in Colomi are already available, to varying extents, in commercial markets. Furthermore, many of them are produced in other regions of Bolivia, so a single year’s poor harvest in Colomi, or any other region, is unlikely to wipe out production entirely.

For products, which are generally sold fresh to the end-consumer, relying on a market intermediary is already a common practice. For example, in the cases of oca and papalisa, intermediaries often purchase tubers in bulk at Colomi’s weekly market, subsequently re-selling them in other regions. Neither product is sold with a high degree of processing, so the crucial factors enabling markets to substitute local consumption are likely to be storage conditions (preventing rot or loss to pests) and transport costs (maintaining a price accessible to low-income households). The processing steps required to make them palatable are simple and can be performed at or after the end-point of sale.

For NTCs requiring more elaborate manipulation, the substitution of household-scale processing with market-oriented industrial processing presents greater complexities. On one hand, a shift to industrial processing might have the positive effect of addressing food safety issues. For example, *charke de llama* was until recently considered taboo by urban consumers, due both to ethnic discrimination and concerns about sanitation and disease (82). Although llama meat was commonly consumed in urban centers during the first author’s fieldwork, research interlocutors also shared stories of encountering poorly processed or spoiled *charke* in commercial outlets. One research informant suggested that this risk might be overcome by promoting the processing of llama-meat off-farm, in centralized slaughterhouses, in order to facilitate hygiene standards suitable for long-distance transport and storage.

On the other hand, substituting household processing with industrial production would also require the substitution of the environmental resources – water, heat, and humidity – that are necessary to prepare *chuño*, *charke*, and *tarwi*. It is theoretically feasible to manipulate these conditions in a controlled environment. As a case in point, one returned Bolivian migrant told the story of having prepared *chuño* while living in Europe by exposing potatoes first to freezing winter temperatures and then to the heat of his home radiator. However, reproducing such processes at a larger scale, in order to fully satisfy consumer markets, would imply major investments of energy, increasing the product’s cost. Furthermore, in the Valley of Cochabamaba, where water shortages are frequent, and water access is highly political (83, 84), it is questionable whether the abundant, clean, cold running water necessary to process *chuño* and *tarwi* would be readily available, accessible, or politically justifiable at an industrial scale.

Despite these barriers, many of these products are already available in Cochabamba’s urban markets and are sold in bulk quantities. Although some of this market is supplied by smallholder farmers, some industrial processing efforts are underway, including the production of potato chips from native varieties in Colomi, facilitated by Fundación PROINPA (68). Similar processing barriers have also been overcome in the case of another native crop; for quinoa, technologies for removing bitter saponins, which historically involved heavy manual labor, have been substituted similarly effective mechanized processes (65). Additional research on the existing value chains for other NTCs crops currently sold in urban markets, and on consumer demand, is necessary to fully assess the potential of industrial or centralized processing to provide an alternative that would both support agrobiodiversity conservation, and maintain the accessibility of indigenous foods to low-income, nutritionally vulnerable consumer groups.

## Conclusion: Dueling Epistemologies in Indigenous Food Systems and Climate Change Research

This article has presented information about an indigenous food system, based on native and traditional crops, in Colomi, Bolivia. In this site, the characteristics not only of agricultural production but also of crop and livestock processing and preservation present important vulnerabilities to the weather and climatic changes currently being observed in the region. These observations suggest potential limitations to the viability of household processing systems that remove unpalatable toxins, and allow for post-harvest preservation, as well as the possibility of the loss of agrobiodiversity from the local food system.

Although this example is based on a single case, it raises issues worthy of study in other world regions. To the extent that what we have termed “indigenous food systems” share common characteristics, they may be vulnerable to climate change in ways that differ from industrial agriculture. For example, such crops may be cultivated in environments which are especially affected by climatic changes, such as high-mountain regions, arid lands, or areas susceptible to sea-level rise.^3^ When these crops are primarily utilized for subsistence consumption, or are cultivated by populations that are socially or economically marginalized, their loss may imply immediate negative impacts on the nutrition and food security of the farmers who cultivate them. Such impacts may differ, in terms of speed, scope, or nutritional implications, from the impacts of climate change on households already more completely embedded in industrially driven food systems.

Further research on the impacts of climate change on specific indigenous food systems is particularly important given that it will require different scales and scopes of data input than many existing models. Models of the impacts of climate change on agriculture and health tend to be based on large-scale, aggregate data covering large geographic areas, rather than site-specific scenarios. For example, projections of climate change impacts on agriculture rely primarily on data from well-studied cropping systems, such as rice, wheat, and corn, and soybeans (5–7). Given that climate change models are “data hungry,” the reliance on projections from these well-studied crops is intuitive. However, while smallholder farmers are likely to directly experience the impacts of climate change, their production conditions and cropping systems are very different from those of industrial agriculture and may be far more challenging to model (86).

Projections of climate change impacts on environmental health and food security also tend to be guided by large-scale, aggregate frameworks, with the purpose of identifying major global trends. Such frameworks provide overarching guides to the predicted health impacts of increased temperatures and reduced yields and productivity (9). However, authors acknowledge the challenges inherent in predicting specific health outcomes, such as changes in patterns of malnutrition, due to the complexity of the factors and influences that may lead to the development of poor health at the individual level (87).

The lack of site-specificity in this body of research is problematic in particular when considering nutritional outcomes for individuals cultivating or otherwise relying on indigenous food systems. Such food systems generally rely on non-industrial crops not included in aggregate models and are cultivated primarily by smallholder farmers. Furthermore, particularly in regions of the world experiencing rapid urban–rural migration, people living in such systems may be undergoing a rapid “nutrition transition” (88), entailing a shift from calorie-limited food environments to environments with a high prevalence of fat, sugar, and meat. The increasing prevalence of such conditions is implicated in rising rates of non-communicable diseases, such as heart disease and diabetes (88, 89).

In ethnically indigenous populations, such changes may have additional unforeseen consequences. Research has recently begun to identify mechanisms by which populations of shared genetic backgrounds may respond differently to the intake of particular foods (90) or environmental toxins (91). In this context, NTCs may represent an important resource both for improving nutritional outcomes and helping consumers to find culturally meaningful understandings of the causes and treatments of diet-related disease (92). Although aggregate analyses of climate change, agriculture, and nutrition are important for policy and planning, they are insufficient to address these complex patterns of demography, genetics, diet, and culture.

A significant body of existing literature has explored the dynamics, distribution, and maintenance of on-farm agrobiodiversity, of the type that is here denoted by the term “indigenous” food systems (24, 31–42). Related inquiries have investigated the articulation of small-scale, biodiverse subsistence farming with commercial markets and larger patterns of economic development (43, 45, 46, 66, 67), noting that even in the context of economic and technological change, farmers’ local varieties and minor crops can play important roles in household and regional economics. Similarly, an emerging literature details the importance of such crops and foods for the food security and nutrition to households that cultivate them, and consumers purchasing them in markets (93–97).

Nonetheless, newly developing environmental contexts driven by climate change beg new hypotheses for future research. There is a need for synthetic studies that explicitly describe and explore the relationships linking the climate/weather environment, the maintenance and use of agricultural biodiversity, the cultural and food preferences of specific population groups, the coping strategies that groups and individuals use to respond to climate and weather changes, and the health outcomes that these factors synergistically generate. Such research may build on the robust body of existing studies in ecology, agronomy, and anthropology, but will require new interdisciplinary approaches, including collaboration with the health, nutritional, and atmospheric sciences. New and creative methodological tools may also be necessary to adequately study systemic relationships linking environment, culture, and health. Due to the immediacy of the impacts of climate change on indigenous foods and foodways, indigenous food systems merit urgent attention on the part of researchers and practitioners of agricultural development, food security, and environmental health.

## Author Contributions

AKS did primary data collection, analysis, and writing of the article; XCF and RGH contributed data, analysis, and editing; DH advised data collection and contributed to analysis and writing/editing.

## Conflict of Interest Statement

The authors declare that the research was conducted in the absence of any commercial or financial relationships that could be construed as a potential conflict of interest.

## Footnotes

^1^Agricultural systems like those we term “indigenous food systems” are well-studied in both the social science and the natural science literatures. The economic and social configurations of such systems have been robustly described in analyses of smallholder agriculture, and peasant agriculture (31–33). The diverse ecological components of these systems have also been well examined, under the banners of crop diversity (34), infra-specific diversity (24), agrobiodiversity (35), agrodiversity (36, 37), or agroecology (38, 39). The identity and rights of people who live within these systems have been considered under the terms farmers’ rights (40, 41) or food sovereignty (42). We propose the term “indigenous food systems” not to supplant any of these existing frameworks, but to emphasize relationships linking production, marketing, consumption, and health. Our conceptualization of the “system” explicitly links the production practices used in biodiverse agriculture with consumption, both at the level of rural households and by urban consumers sourcing food through markets, and in turn links consumption practices to health outcomes, *via* the influences of food security and nutrition.

^2^The cultivators and food consumers in such systems may identify themselves as ethnically indigenous, and their personal and family histories may be important for understanding the health impacts of changing nutritional patterns. However, not all farmers using “indigenous” cultivation techniques would readily identify themselves as ethnically indigenous. Contemporary anthropological understandings of indigeneity stress that the term might better be read as a political positioning of a speaker vis-à-vis his or her audience, rather than a specific claim to heritage or genetics (47). Hence, for this analysis, focusing primarily on biophysical aspects of agriculture, environment, and health, we use the term “indigenous” to refer to a constellation of seed materials and cultivation, preparation, trade, or consumption practices, which differ from those commonly observed in industrial-scale agriculture.

^3^Such areas may also be important for the conservation of crop genetic diversity, since geographical areas of high abiotic stress tend to be associated with high levels of genetic polymorphisms (85).
